# Creatine supplementation in young men under resistance versus non-resistance training: a systematic review and meta-analysis of strength, performance, and lean mass

**DOI:** 10.3389/fnut.2026.1800546

**Published:** 2026-04-08

**Authors:** Jinfa Gu, Yan Li, Jialing Xiao, Yu Zhang

**Affiliations:** 1 School of Stomatology, Qilu Medical University, Zibo, China; 2 Exercise & Sports Science Programme, School of Health Sciences, Universiti Sains Malaysia, Kubang Kerian, Kelantan, Malaysia; 3 Department of Pharmacy, Ezhou Central Hospital, Ezhou, Hubei, China; 4 College of Nursing and Health, Jiujiang Polytechnic University of Science and Technology, Gongqingcheng, China

**Keywords:** anaerobic performance, body composition, creatine, meta-analysis, resistance training, supplementation

## Abstract

**Background:**

Creatine is a highly marketed ergogenic aid that has strengthening and high-intensity training effects. However, past meta-analyses have often grouped together heterogeneous training modalities, and it was not known if the training context has a modifying effect on body composition and performance.

**Methods:**

This systematic review and meta-analysis pooled RCT evidence in healthy men aged 18–30 years old to quantify the effects of creatine supplementation in terms of body composition, maximal strength, and exercise performance. All the databases were searched up to 1 October 2025, and 37 eligible trials were discovered. The context of training was prespecified—RT vs. non-RT, and used as the main comparison. Pooled estimates were made using random effects models for FFM, LBM, and Wingate peak and mean power, CMJ, and 1RM outcomes. The exploratory subgroup analyses were done to investigate whether training condition and intervention duration moderated the effects.

**Results:**

The number of trials that were considered according to the inclusion criteria was 37. When using RT, creatine supplementation led to significant gains in FFM (+3.39 kg) and LBM (+2.70 kg), but not to significant gains in non-RT conditions. Wingate peak and mean power both increased in both contexts (peak power +71.27 W; mean power +39.69 W), with no evidence that training context modified these results. CMJ showed a pooled improvement of 2.70 cm; however, this estimate should be interpreted with caution due to high heterogeneity (*I*^2^ = 89%). Exploratory subgroup analyses by intervention duration should be interpreted cautiously because of the small number of studies and high heterogeneity.

**Conclusion:**

The supplementation with creatine leads to an increase in anaerobic power regardless of the training environment, but the gains in body composition depend on parallel RT. In practice, creatine in association with RT is recommended for lean mass gains, while either anaerobic performance benefit could be obtained in different training modalities.

**Systematic review registration:**

The study was prospectively registered in PROSPERO (CRD420261283973). The registration URL is: https://www.crd.york.ac.uk/prospero/display_record.php?RecordID=1283973.

## 1 Introduction

Creatine is an inborn nitrogen-containing compound, of which approximately 95% is present in the skeletal muscle; smaller quantities are found in tissues such as the brain, myocardium, and testes (1, 2). Regular dietary supplementation accounts for 60–80% of the overall body creatine and phosphocreatine (PCr), and exogenous creatine nutritional supplementation can further increase intramuscular creatine and PCr levels by between 20 and 40% (3, 4). The phosphocreatine-creatine kinase system is involved in the rapid extraction of a phosphate group of adenosine diphosphate to restore adenosine triphosphate (ATP) during high-power output and demands short-duration, high-intensity muscle contractions (5–7). It is on this mechanistic explanation that creatine supplementation is largely used as an ergogenic aid within the sport and exercise setting.

Randomized controlled trials and meta-analyses have been performed to study the effects of creatine on maximal strength, anaerobic performance, and body composition. For strength outcomes, previous syntheses are quite consistent in showing that benefits from creatine supplementation are greatest when creatine supplementation is coupled with structured resistance training (RT) (8, 9). This type of pattern is often attributed to improvements in training quality, tolerance to high-intensity loading, and recovery that have been shown with creatine, which may in turn lead to enhanced neuromuscular adaptations to training (10).

Creatine impacts body composition, with a wider variety of effects, particularly on the outcome of lean mass. Several systematic reviews and meta-analyses have found modest increases in lean body mass or fat-free mass; however, some have also found large between-study variability (11, 12). Differences in participants, supplementation protocols, training modalities, and assessment methods have all been put forward as contributing to this heterogeneity (13, 14). Training modality is often studied only as part of *post hoc* subgroup analyses, often as part of reviews including mixed sex and broad age ranges. As such, both the specificity and translational relevance of conclusions are limited. One such source is the training context, which is especially relevant, as it is through this context that the mechanisms underlying the expected adaptations operate and, at the same time, are actionable within the framework of program design.

Training context is a potentially likely effect modifier of creatine supplementation. The nature of the training stimulus will dictate the targeted muscle groups, loading conditions, cumulative training volume, and adaptive pathways used (15, 16). RT, in particular, offers a dramatic and progressive hypertrophic stimulus that might enable the metabolic benefits of creatine to result in measurable increases in lean mass (17, 18). Conversely, however, non-resistance training (non-RT) situations involve a wide variety of modalities whose loading patterns have a wider range of heterogeneity and can suppress or dilute such effects (19, 20). One outcome of this is that anaerobic outcomes are potentially different between modalities due to differences in neuromuscular demands and work-rest structures, which determine how enhanced availability of PCr is translated into measurable high-intensity performance.

Despite the relevance of training context, the existing evidence syntheses have several limitations. First, most reviews have confounded different groups of individuals and training approaches, downplaying the inferential debate on specific groups (11, 17). Second, the training context is often treated as a secondary or exploratory factor rather than being taken as a prespecified analytical framework, which makes inference on moderation weak (21). Third, performance and body composition outcomes are frequently evaluated in isolation, limiting integrated interpretation for exercise prescription (22). Thus, it is not established whether training context moderates creatine’s effects on performance and lean mass in young men.

This systematic review and meta-analysis synthesized RCTs in healthy men aged 18–30 years. It evaluated the effects of creatine supplementation on maximal strength, anaerobic performance, and lean mass outcomes (LBM and FFM). The training context was prespecified as RT versus non-RT and served as the primary analytical framework to test whether the training stimulus moderates creatine’s effects. Where data allowed, exploratory subgroup analyses were conducted to investigate heterogeneity while limiting the likelihood of spurious associations.

## 2 Methods

### 2.1 Registration and reporting standards

This systematic review and meta-analysis followed PRISMA reporting guidelines and the methodological recommendations of the Cochrane Handbook (23). The protocol was prospectively registered in PROSPERO (CRD420261283973) (24). Primary outcomes and the training context–stratified analytical framework (RT vs. non-RT) were prespecified.

### 2.2 Search strategy

Two reviewers independently searched PubMed, Web of Science, Scopus, Embase, the Cochrane Library, and SPORTDiscus from inception to 1 October 2025 (Supplementary File 1). The search combined controlled vocabulary, such as MeSH and Emtree terms, with free-text terms for creatine supplementation and RCT filters, such as random*, trial*, and placebo. To reduce the risk of missed studies, reference lists of all included articles were screened manually. Only full-text articles published in English were eligible, with no restriction on publication year.

### 2.3 Study selection and screening process

All records were imported into reference management software, and duplicates were removed. Study selection followed a two-stage process based on prespecified inclusion and exclusion criteria (25). Screening was performed independently by two reviewers, with disagreements resolved through discussion or adjudication by a third reviewer.

### 2.4 Eligibility criteria

#### 2.4.1 Inclusion criteria

*Participants:* Healthy young men aged 18–30 years. Only studies with exclusively male samples were eligible.

*Intervention:* Creatine supplementation at any dose and regimen. A clearly defined training stimulus during the intervention period was required.

*Comparators:* A creatine-free control condition, including placebo or no creatine supplementation. When an exercise intervention was implemented, the control group was required to follow the same training program as the intervention group, with training frequency, intensity, periodization, and supervision matched as closely as possible. Apart from creatine, conditions were kept comparable where feasible.

*Outcomes:* Studies had to report at least one of the following primary outcomes with extractable data suitable for pooling: (1) maximal lower-body strength, such as one-repetition maximum (1RM) or an equivalent maximal strength test, including leg press or squat; (2) countermovement jump (CMJ) performance; (3) Wingate power indices, including peak and/or mean power; (4) lean mass outcomes, specifically LBM or FFM, with the assessment method recorded.

*Study design:* Randomized controlled trials.

#### 2.4.2 Exclusion criteria

Studies were excluded if they were nonrandomized or lacked a control group; did not include exclusively male participants; enrolled participants outside the 18–30 year range or did not provide sufficient information to confirm eligibility; involved clinical or other special populations; failed to report outcome data usable for meta-analysis and such data could not be obtained after contacting authors; or included important cointervention differences between groups beyond creatine that could not be reasonably evaluated (such as an additional supplement provided only to one group).

During data extraction, included trials were classified by training context as RT or non-RT for stratified analyses.

### 2.5 Data extraction and data handling

Two reviewers independently extracted data using a standardized form, including author and publication year, sample size, participant age and training status or competitive level, creatine protocol (dose, duration, and whether a loading phase was used), comparator details, training content during the intervention and the corresponding training-context classification (RT or non-RT), and extractable data for the prespecified outcomes. Training context (RT vs. non-RT) was defined *a priori*. RT was defined as structured, progressive exercise using external resistance aimed at improving strength and/or hypertrophy. Trials were classified as non-RT if the primary training stimulus did not meet this definition, based on the predominant training modality described in each study. Discrepancies were resolved through discussion and, when necessary, adjudication by a third reviewer. To avoid duplicate weighting, each study contributed at most one effect estimate per prespecified outcome. Lean mass outcomes were extracted as reported in the original trials; therefore, LBM and FFM were not combined, and the assessment method was recorded for each. For all continuous outcomes, post-intervention values (means and standard deviations) were extracted and used for meta-analysis. Change scores were not employed.

### 2.6 Risk of bias and methodological quality assessment

Risk of bias was evaluated using the Cochrane Risk of Bias 2 (RoB 2) tool (26). RoB 2 covers five domains: the randomization process, deviations from intended interventions, missing outcome data, outcome measurement, and selection of the reported result. Each domain was rated as low risk, some concerns, or high risk, and an overall judgment was assigned accordingly. Two reviewers independently assessed risk of bias; discrepancies were resolved by consensus and, when necessary, adjudication by a third reviewer. Findings were summarized graphically.

### 2.7 Certainty of evidence (GRADE)

The certainty of evidence for each outcome was evaluated using the Grading of Recommendations Assessment, Development and Evaluation (GRADE) framework (27). Evidence was rated as high, moderate, low, or very low across five domains: risk of bias, inconsistency, indirectness, imprecision, and publication bias. As all included studies were randomized controlled trials, certainty started at high and was downgraded where appropriate. Two reviewers assessed GRADE independently, with disagreements resolved by consensus.

### 2.8 Statistical analysis

All meta-analyses used random-effects models to account for anticipated between-study variation in participant characteristics, training protocols, and testing methods (28). Continuous outcomes were pooled as mean differences (MDs) with 95% confidence intervals (CIs). Because outcome units were consistent across studies, MDs were used rather than standardized mean differences (SMDs) to preserve interpretability in the original units. Meta-analyses were conducted only when outcomes were measured on comparable scales.

Statistical heterogeneity was quantified using *I*^2^ and interpreted alongside differences in study design, training context, and outcome assessment (29). *I*^2^ values of approximately 25, 50, and 75% were considered to represent low, moderate, and high heterogeneity, respectively.

The primary analyses were stratified by training context (RT vs. non-RT). When both strata contained a sufficient number of studies, effects were pooled within each stratum, and between-group differences were tested to evaluate potential moderation by training context.

Sensitivity analyses were conducted with the use of a leave-one-out approach, where each study was removed individually, and its influence was determined on the pooled estimates. Fixed-effect and random-effects models were also compared to test for robustness.

If an outcome was informed by at least 10 studies, publication bias was assessed using funnel plots and, where possible, Egger’s regression test (30). A two-sided *p*-value of <0.05 was considered statistically significant. Statistical analyses were performed using R (version 4.3.3) via RStudio, primarily employing the meta package for data pooling and the tidyverse for data handling and visualization.

## 3 Results

### 3.1 Study selection

**Figure 1:**
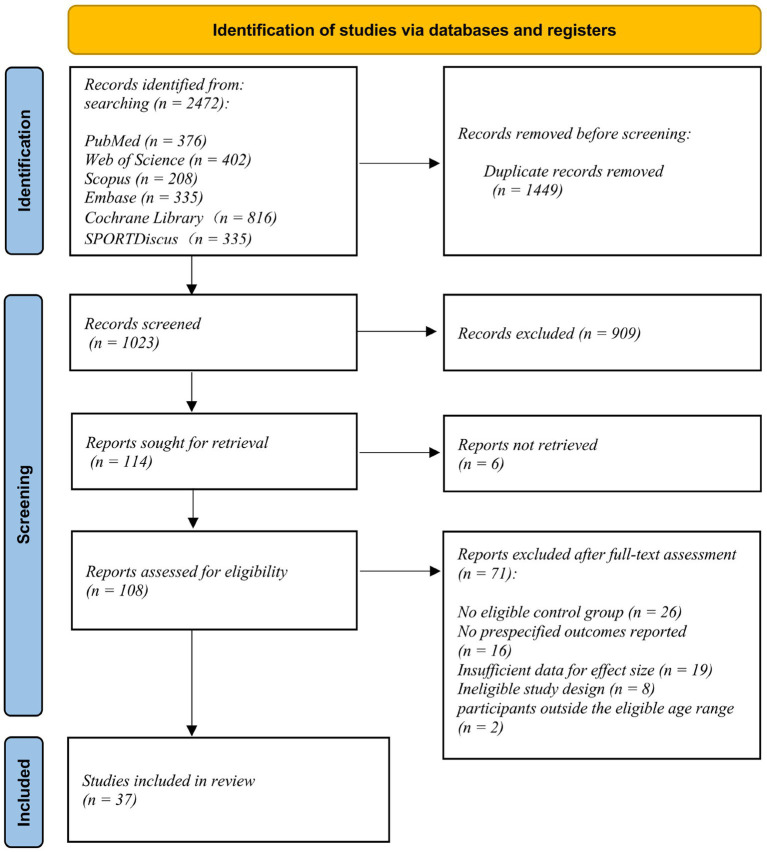
PRISMA flow diagram illustrating the study selection process for the systematic review and meta-analysis.

A total of 2,472 records were identified through database searching. After removal of 1,449 duplicates, 1,023 records were screened, of which 909 were excluded. Full-text reports were sought for 114 records; six reports could not be retrieved, leaving 108 reports assessed for eligibility. Of these, 71 reports were excluded for the following reasons: lack of an eligible control group (*n* = 26), absence of prespecified outcomes (*n* = 16), insufficient data to calculate effect sizes (*n* = 19), inappropriate study design (*n* = 8), and participants outside the eligible age range (*n* = 2). Ultimately, 37 studies were included in the review (see Figure 1).

### 3.2 Study characteristics

A total of 37 such RCTs were included, consisting of 24 studies in RT settings and 13 in non-RT settings (14, 31–66). Not all outcomes were reported in some studies, but in every study, at least one prespecified outcome was noted. Mean ages were between 18.5 and 29.5 years, and training status ranged from sedentary or untrained to welltrained, competitive, and elite athletes. RT was the most common modality (about 65% of studies), with other protocols that included team sports (rugby or American football, soccer, handball, and basketball), individual sports (canoeing or rowing and sprinting), cycling-based protocols (including Wingate testing), and those related to strength- or power-related performance assessment.

Most supplementation protocols used either a loading phase followed by a maintenance phase or a fixed daily dose. The most common loading regimen was 20 g/day, administered in divided doses for 5–7 days, followed by a maintenance dose typically ranging from 2 to 10 g/day. Alternative approaches included body mass–based dosing (approximately 0.07–0.3 g/kg/day), higher fixed doses (25–35 g/day), and polyethylene glycol–bound creatine (1.25–2.50 g/day). Intervention duration ranged from 4 days to 12 weeks, with most trials lasting 4–10 weeks.

Control conditions most frequently consisted of carbohydrate-based comparators (such as glucose, dextrose, maltodextrin, or sucrose-based beverages) or inert placebos (such as cellulose or silica). A small number of studies used protein or carboxymethylcellulose as the comparator.

The primary outcomes included maximal strength (one-repetition maximum (1RM) in the squat or leg press), anaerobic or explosive performance (Wingate power outcomes and CMJ height), and body composition indices (FFM and LBM) (see Table 1).

**Table 1:** Characteristics of included studies.

Study citation	Mean age (years)	Sport/exercise modality	Subject level	Intervention protocol (dosage and frequency)	Duration	Control	Outcome measures
Ahmun et al. (31)	20.6	Rugby union	Highly trained	20 g/d (4 × 5 g)	5 days	Dextrose	Wingate power
Arciero et al. (32)	21	Resistance training	Healthy active (resistance-untrained)	20 g/d (5 days) + 10 g/d (23 days)	28 days	Dextrose	1RM Leg Press, FFM
Becque et al. (33)	21.5	Arm flexor strength training	≥1 year of weight training experience	Loading: 20 g/d (5 g × 4) for 5 days; maintenance: 2 g/d	6 weeks	Flavored sucrose drink	FFM
Bemben et al. (34)	19.2	American football	NCAA Division I athletes (redshirt)	20 g/d (5 days) + 5 g/d (Maintenance)	9 weeks	Glucose	1RM squat, Wingate power, LBM
Bonilla et al. (35)	26.6	Cluster-set resistance training	Resistance-trained (>2 years experience)	0.1 g/kg/d (post-workout)	8 weeks	Protein	1RM squat, CMJ, LBM
Camic et al. (36)	22.1	Anaerobic Performance Tests	Untrained (in resistance exercise)	1.25 or 2.50 g/d PEG-creatine (1 dose/d)	28 days	Cellulose	CMJ, FFM
Cribb et al. (37)	24	Bodybuilding	Recreational male bodybuilders	1.5 g/kg/d Supplement (~0.3 g/kg load + 0.1 g/kg Maint)	11 weeks	Glucose	1RM squat, FFM
Del Favero et al. (38)	24	Strength/power tests	Untrained (not engaged in RT)	20 g/d (2 × 10 g)	10 days	Dextrose	1RM squat, LBM
Earnest et al. (39)	29.5	Bench press and Wingate bike tests	~11 years of training experience	20 g/d (5 g × 4) during the supplementation period	28 days	Glucose placebo	FFM
Griffen et al. (40)	21.6	Cycling (Wingate)	Well-trained men	20 g/d (4 × 5 g)	7 days	Placebo	Wingate power
Havenetidis et al. (41)	29.4	Cycling (Wingate)	Sprint Trained Males	10–35 g/d (divided doses)	4 days	Placebo	Wingate power
Herda et al. (42)	21	Resistance exercise/Wingate	Recreationally active	5 g/d (1 dose/d)	30 days	Placebo	Wingate power
Hoffman et al. (43)	19	American football	Collegiate athletes	10.5 g/d (2 doses/d)	10 weeks	Placebo	1RM squat, Wingate power
Izquierdo et al. (44)	22	Handball	Trained athletes	20 g/d (4 × 5 g)	5 days	Placebo	1RM squat, CMJ
Javierre et al. (45)	22.9	Running (sprints)	Physically active	20 g/d (4 × 5 g)	5 days	Placebo	CMJ
Kaviani et al. (46)	23	Resistance training	Sedentary/inactive	0.07 g/kg/d (2 doses/d)	8 weeks	Placebo	1RM leg press
Kelly et al. (47)	26.8	Powerlifting	Competitive	20 g/d (Load) + 5 g/d (Maint)	26 days	Glucose	LBM
Kilduff et al. (48)	24	Isometric bench-press	At least 2 years of structured training experience	20 g/d (10 g × 2) mixed with 180 g dextrose for 5 days	5 days	200 g/d glucose polymer	FFM
Law et al. (49)	23.1	Basketball	Trained athletes	20 g/d (4 × 5 g)	5 days	Maltodextrin	1RM squat, Wingate power
Mujika et al. (50)	20.3	Soccer	Highly trained	20 g/d (4 × 5 g)	6 days	Maltodextrin	CMJ
Noonan et al. (51)	19.4	Weight training and speed drills	NCAA division II football team	Loading: 20 g/d (5 g × 4) for 5 days; Maintenance: 100 or 300 mg/kg FFM	8 weeks	Dextrose placebo	FFM
Nunes et al. (14)	22.7	Resistance training	Resistance trained	0.3 g/kg/d (Load) + 0.03 g/kg/d (Maint)	8 weeks	Maltodextrin	LBM
Peeters et al. (52)	21.2	Resistance training	Experienced (>2 yrs)	20 g/d (Load) + 10 g/d (Maint)	6 weeks	Maltodextrin	1RM leg press, LBM
Saremi et al. (53)	23.4	Resistance training	Healthy untrained	0.3 g/kg/d (Load) + 0.05 g/kg/d (Maint)	8 weeks	Cellulose	1RM leg press, LBM
Stone et al. (54)	18.5	American football	Collegiate athletes	0.22 g/kg/d (3 doses/d)	5 weeks	Placebo (Silica)	1RM squat, CMJ, Wingate power, LBM
Stout et al. (55)	19.6	American football	Collegiate athletes	21 g/d (Load) + 10.5 g/d (Maint)	8 weeks	Glucose (CHO)	CMJ
Syrotuik et al. (56)	22.1	Resistance training	Resistance trained	0.3 g/kg/d (Load) + 0.03 g/kg/d (Maint)	37 days	Placebo	1RM Leg Press
Taylor et al. (57)	21.3	Resistance training	Resistance trained	5 g/d (1 dose/d)	8 weeks	Placebo	1RM Leg press, Wingate power, LBM
Trexler et al. (58)	21.2	Resistance training	Recreationally Trained	5 g/d (1 dose/d)	28 days	Placebo	1RM Leg Press
van Loon et al. (59)	20.6	Repeated supramaximal sprint and endurance cycling	No history of regular exercise training	Loading: 20 g/d for 5 days; Maintenance: 2 g/d for 37 days	6 weeks	Placebo without creatine	FFM
Volek et al. (60)	23.4	Resistance training	Resistance trained	25 g/d (Load) + 5 g/d (Maint)	12 weeks	Placebo	1RM squat, FFM
Volek et al. (61)	20.3	Resistance training (overreaching)	Resistance Trained	0.3 g/kg/d (Load) + 0.03 g/kg/d (Maint)	4–5 weeks	Placebo	1RM squat, LBM
Wang et al. (63)	21.1	Complex training (squat/jump)	University Athletes	20 g/d (Load) + 2 g/d (Maint)	30 days	CMC	1RM squat
Wang et al. (62)	20.2	Kayak/canoe	University athletes	20 g/d (4 × 5 g)	6 days	CMC	1RM squat, CMJ, FFM
Wilder et al. (64)	19.3	American football	Collegiate athletes	20 g/d (Load) + 5 g/d (Maint)	10 weeks	Glucose polymers	1RM squat, FFM
Willoughby & Rosene (65)	20.4	Resistance training	Untrained males	6 g/d (1 dose/d)	12 weeks	Dextrose	LBM
Zuniga et al. (66)	22.5	Resistance training	Resistance trained	20 g/d (4 × 5 g)	7 days	Maltodextrin	Wingate power

1RM, one-repetition maximum; CMJ, countermovement jump; FFM, fat-free mass; LBM, lean body mass; RT, resistance training; CHO, carbohydrate; PEG, polyethylene glycol (PEG-creatine); CMC, carboxymethylcellulose; g/d, grams/day; g/kg/d, grams/kg/day. Loading and maintenance denote high-dose initiation followed by a lower-dose phase; post-workout indicates intake immediately after training.

### 3.3 Risk of bias results

**Figure 2:**
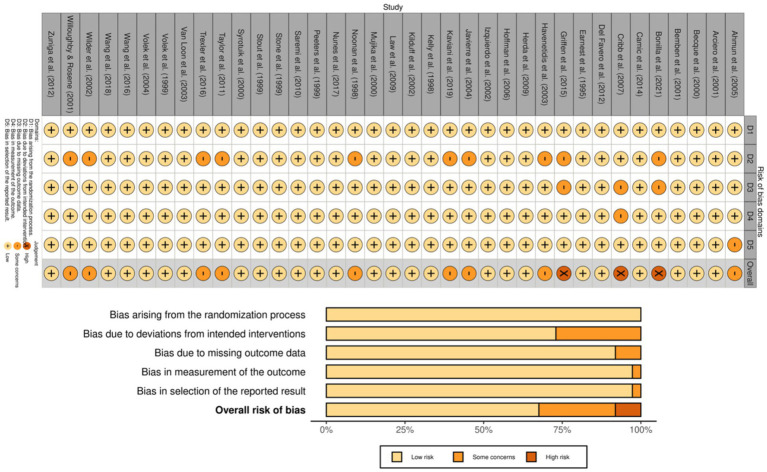
Risk-of-bias assessment of included trials using the Cochrane RoB 2 tool.

Risk of bias was evaluated using the Cochrane RoB 2 tool. Among the 37 trials, 25 (67.6%) were deemed to be at low risk of bias, nine (24.3%) raised some concerns, and three (8.1%) were considered to be at high risk. Some concerns most commonly arose from deviations from intended interventions (D2), with fewer studies flagged for missing outcome data (D3) or selective reporting (D5). Outcome measurement (D4) was regarded as low risk in nearly all trials (36/37), consistent with predominantly objective performance and body-composition outcomes (see Figure 2).

### 3.4 Overall effects

Compared with control conditions, the pooled effect favored creatine supplementation for squat 1RM (16 studies, *N* = 300; MD = 11.9 kg, 95% CI 7.60 to 16.20; *p* < 0.001; *I*^2^ = 43.1%), whereas no significant pooled effect was observed for leg press 1RM (12 studies, *N* = 273; MD = 3.58 kg, 95% CI −7.85 to 15.01; *p* = 0.539; *I*^2^ = 39.1%).

For countermovement jump, the pooled effect favored creatine supplementation (11 studies, *N* = 273; MD = 2.70 cm, 95% CI: 0.18–5.21; *p* = 0.04; *I*^2^ = 89%). The high heterogeneity indicates substantial variability across the included studies, which may reduce the stability of the pooled estimate. For anaerobic performance, significant pooled effects were observed for Wingate peak power (12 studies, *N* = 246; MD = 71.27 W, 95% CI 38.09 to 104.45; *p* < 0.001; *I*^2^ = 13.8%) and Wingate mean power (11 studies, *N* = 218; MD = 39.69 W, 95% CI 15.83 to 63.56; *p* = 0.001; *I*^2^ = 40.5%).

Significant pooled effects favoring creatine supplementation were also observed for FFM (15 studies, *N* = 327; MD = 2.32 kg, 95% CI 0.76 to 3.89; *p* = 0.004; *I*^2^ = 13.6%) and LBM (16 studies, *N* = 344; MD = 1.84 kg, 95% CI 0.53 to 3.16; *p* = 0.006; I2 = 37%).

**Figure 3:**
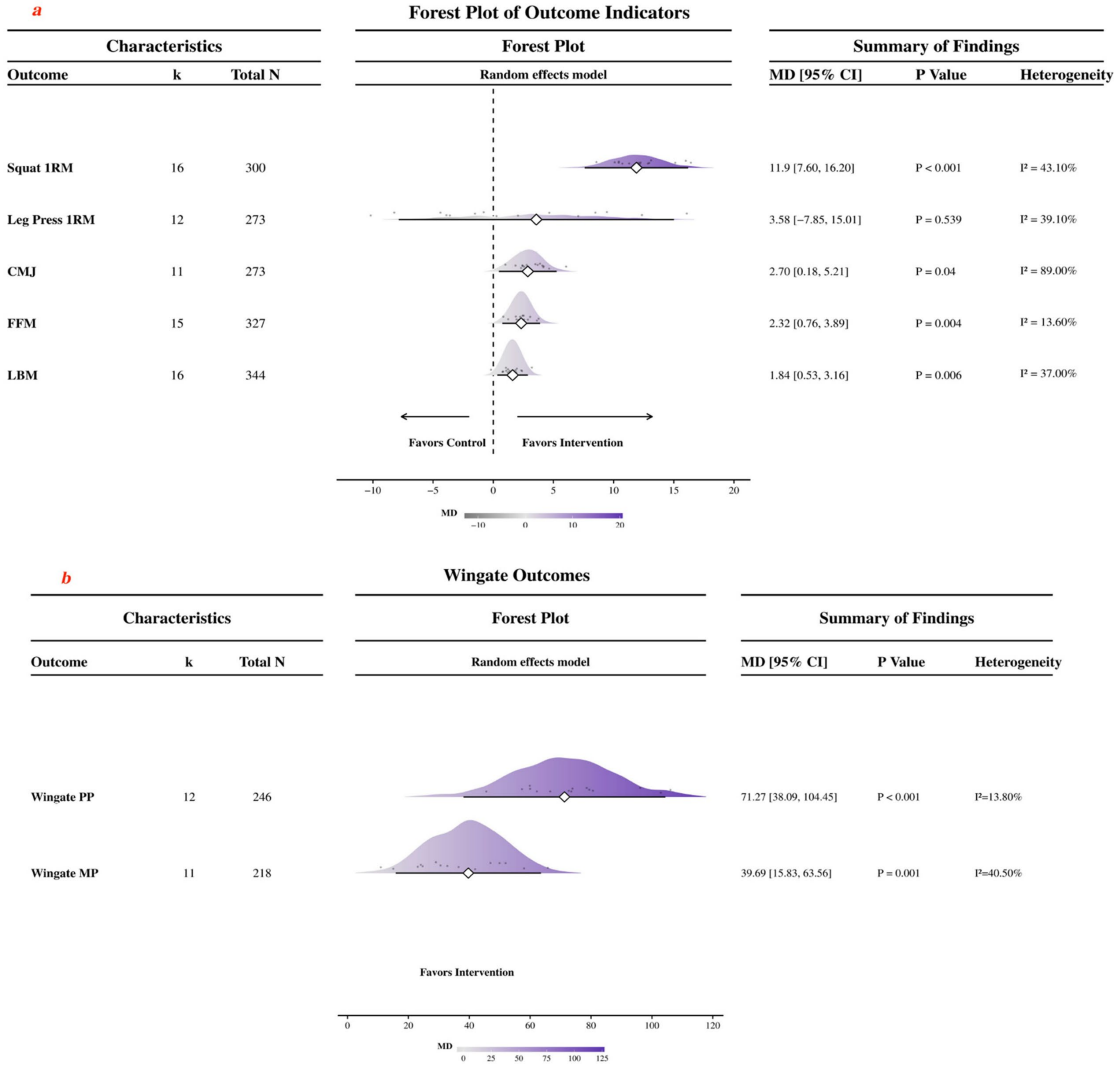
Forest plot of the effects of creatine supplementation on maximal strength, power, anaerobic performance, and body composition. **(a)** Forest plots for squat 1RM, leg press 1RM, CMJ, FFM, and LBM outcomes. **(b)** Forest plots for Wingate peak power and mean power outcomes.

**Figure A1:**
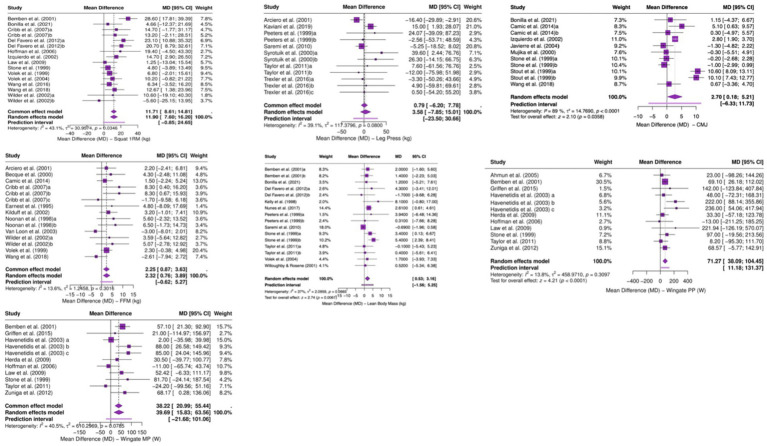
Outcome-specific forest plots for squat 1RM, leg press 1RM, CMJ, Wingate peak power, Wingate mean power, FFM, and LBM.

Forest plots are summarized in Figure 3; the corresponding outcome-specific plots are presented in Figure A1.

### 3.5 Subgroup analysis by training context (RT versus non-RT)

For squat 1RM, significant pooled effects were observed in both RT (12 studies, *N* = 219; MD = 9.58 kg, 95% CI 4.47 to 14.70; *p* < 0.001; *I*^2^ = 42.4%) and non-RT studies (4 studies, *N* = 81; MD = 17.57 kg, 95% CI 11.68 to 23.47; *p* < 0.001; *I*^2^ = 0%), with evidence of a between-subgroup difference (*p* = 0.022; *I*^2^ = 81.0%).

**Figure 4:**
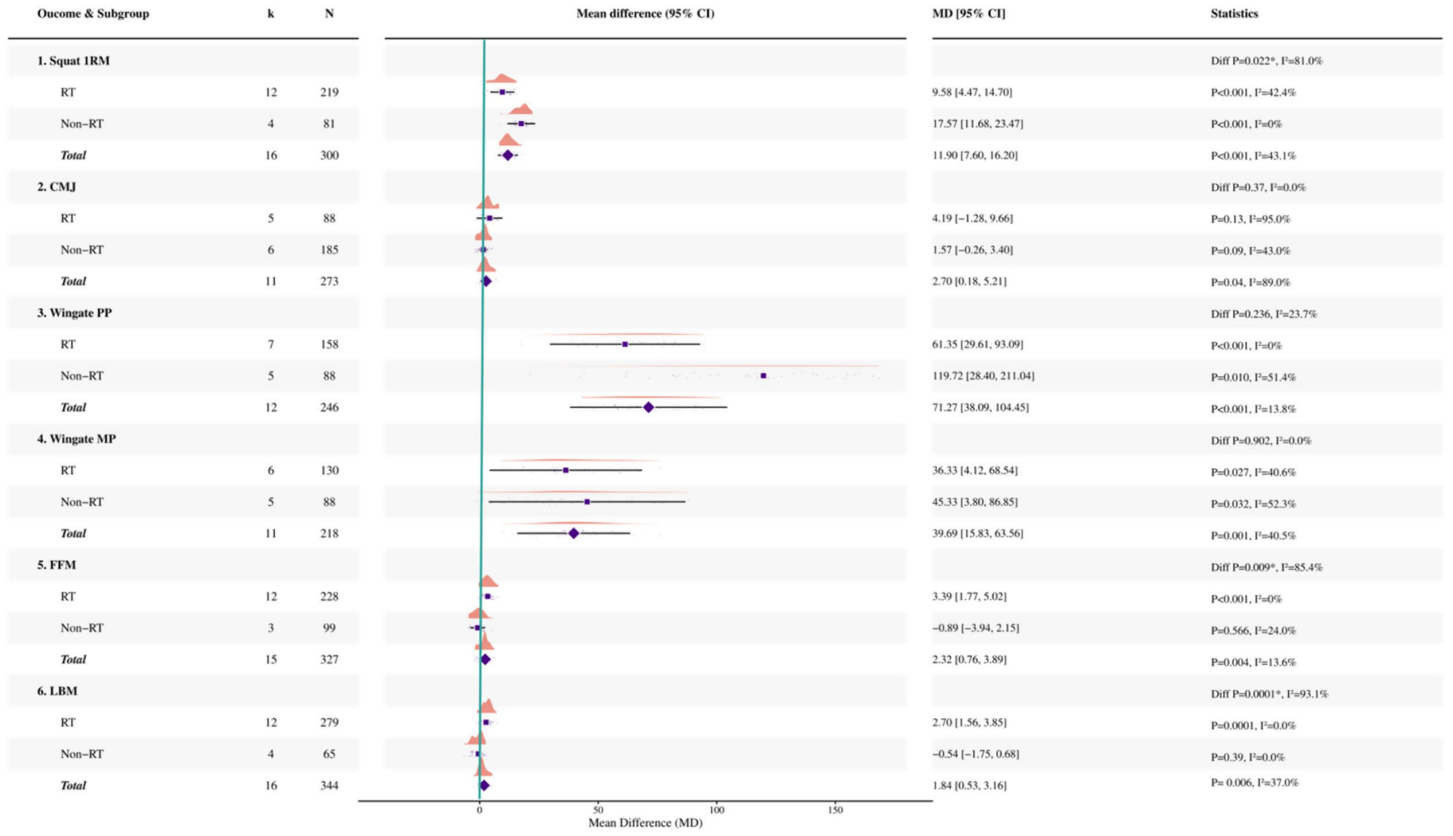
Forest plot of subgroup analyses stratified by RT and non-resistance training (non-RT).

For countermovement jump, the pooled effect was not significant in RT studies (five studies, *N* = 88; MD = 4.19, 95% CI −1.28 to 9.66; *p* = 0.13; I2 = 95%), whereas no significant pooled effect was observed in non-RT studies (six studies, *N* = 185; MD = 1.57, 95% CI −0.26 to 3.40; *p* = 0.09; *I*^2^ = 43%); the between-subgroup difference did not reach conventional statistical significance (*p* = 0.37; *I*^2^ = 0%; see Figure 4).

For Wingate peak power, significant pooled effects were observed in RT (7 studies, *N* = 158; MD = 61.35 W, 95% CI 29.61 to 93.09; *p* < 0.001; *I*^2^ = 0%) and non-RT studies (5 studies, *N* = 88; MD = 119.72 W, 95% CI 28.40 to 211.04; *p* = 0.010; *I*^2^ = 51.4%), with no evidence of a between-subgroup difference (*p* = 0.236; *I*^2^ = 23.7%). For Wingate mean power, significant pooled effects were also observed in RT (6 studies, *N* = 130; MD = 36.33 W, 95% CI 4.12 to 68.54; *p* = 0.027; *I*^2^ = 40.6%) and non-RT studies (5 studies, *N* = 88; MD = 45.33 W, 95% CI 3.80 to 86.85; *p* = 0.032; *I*^2^ = 52.3%), with no evidence of a between-subgroup difference (*p* = 0.902; *I*^2^ = 0%).

For body composition outcomes, a significant pooled effect was observed for FFM in RT studies (12 studies, *N* = 228; MD = 3.39 kg, 95% CI 1.77 to 5.02; *p* < 0.001; *I*^2^ = 0%), but not in non-RT studies (three studies, N = 99; MD = −0.89 kg, 95% CI −3.94 to 2.15; *p* = 0.566; *I*^2^ = 24.0%), with evidence of a between-subgroup difference (*p* = 0.009; I2 = 85.4%). LBM showed a significant pooled effect in RT studies (12 studies, *N* = 279; MD = 2.70 kg, 95% CI 1.56 to 3.85; *p* = 0.0001; *I*^2^ = 0%), whereas no significant pooled effect was observed in non-RT studies (four studies, *N* = 65; MD = −0.54 kg, 95% CI −1.75 to 0.68; *p* = 0.39; *I*^2^ = 0%); the between-subgroup difference was significant (*p* = 0.0001; *I*^2^ = 93.1%).

### 3.6 Subgroup analyses for CMJ

**Figure 5:**
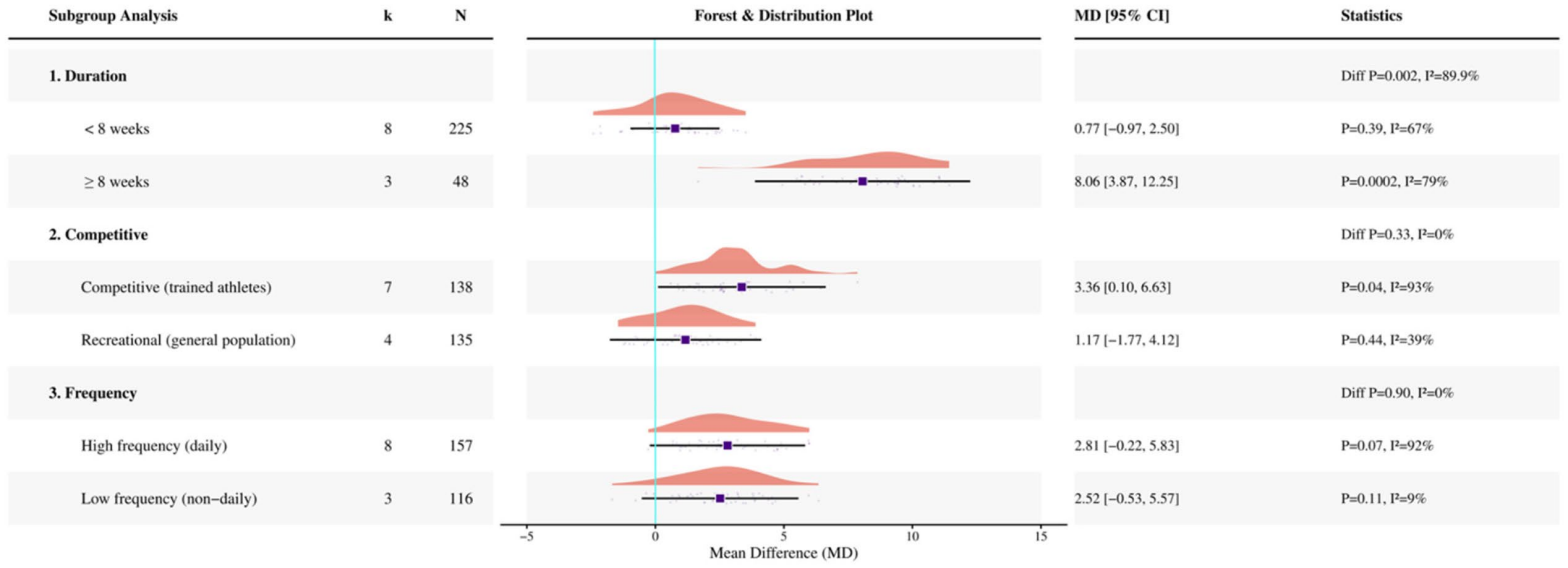
Forest plot of subgroup analyses for CMJ.

When stratified by intervention duration, the pooled effect was not significant in studies lasting <8 weeks (eight studies, *N* = 225; MD = 0.77 cm, 95% CI −0.97 to 2.50; *p* = 0.39; *I*^2^ = 67%), whereas a significant pooled effect was observed in studies lasting ≥8 weeks (three studies, *N* = 48; MD = 8.06 cm, 95% CI 3.87 to 12.25; *p* = 0.0002; *I*^2^ = 79%); a between-subgroup difference was observed (*p* = 0.002; *I*^2^ = 89.9%; see Figure 5).

When stratified by competitive level, a significant pooled effect was observed in competitive participants (seven studies, *N* = 138; MD = 3.36 cm, 95% CI 0.10 to 6.63; *p* = 0.04; *I*^2^ = 93%), whereas no significant pooled effect was observed in recreational participants (four studies, *N* = 135; MD = 1.17 cm, 95% CI −1.77 to 4.12; *p* = 0.44; *I*^2^ = 39%); no significant between-subgroup difference was detected (*p* = 0.33; *I*^2^ = 0%).

When stratified by supplementation frequency, no significant pooled effect was observed in studies with daily supplementation (eight studies, *N* = 157; MD = 2.81 cm, 95% CI −0.22 to 5.83; *p* = 0.07; *I*^2^ = 92%), whereas no significant pooled effect was observed in studies with non-daily supplementation (three studies, *N* = 116; MD = 2.52 cm, 95% CI −0.53 to 5.57; *p* = 0.11; *I*^2^ = 9%); no evidence of a between-subgroup difference was observed (*p* = 0.90; *I*^2^ = 0%).

### 3.7 Sensitivity analysis

Sensitivity analyses using leave-one-out procedures and fixed-effect versus random-effects models produced consistent pooled estimates, with no single study materially influencing results. This pattern also held for CMJ despite substantial heterogeneity in the main analysis.

### 3.8 Assessment of publication bias

**Figure 6:**
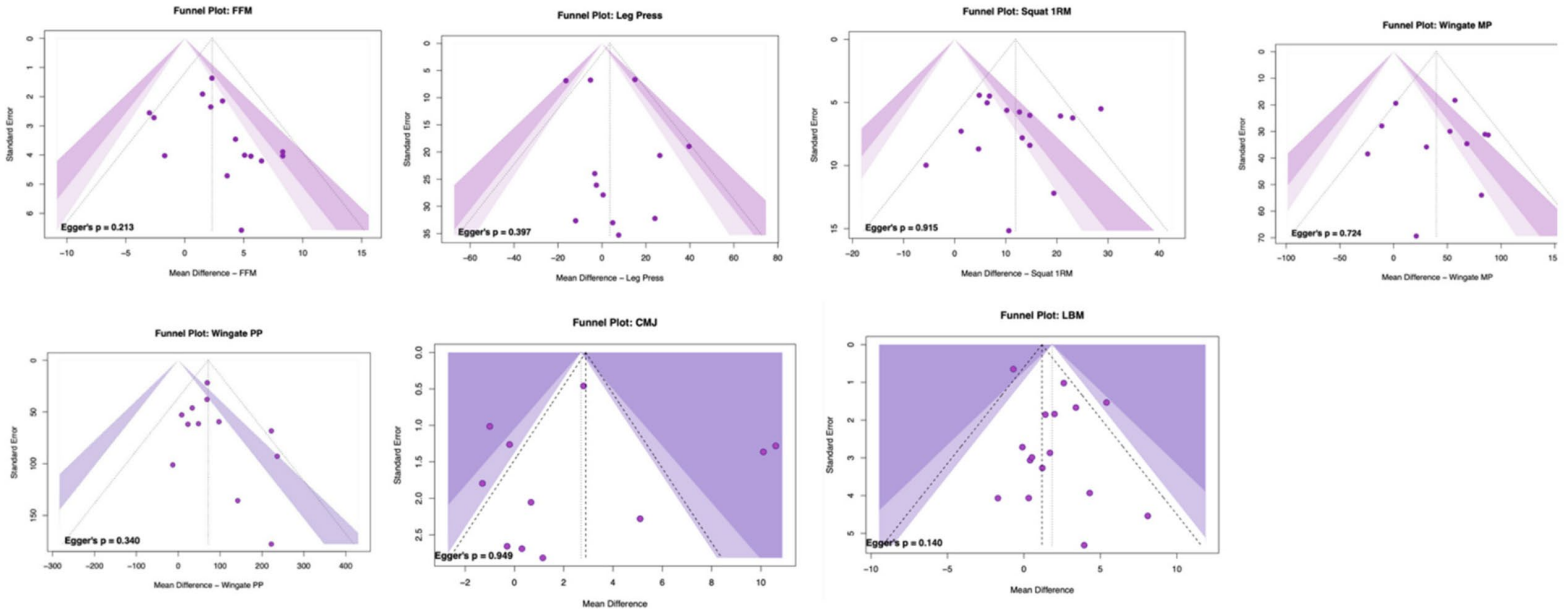
Funnel plots assessing publication bias.

Visual inspection of funnel plots revealed no clear asymmetry, and Egger’s tests were non-significant across outcomes (squat 1RM, *p* = 0.915; leg press 1RM, *p* = 0.397; CMJ, *p* = 0.949; Wingate peak power, *p* = 0.340; Wingate mean power, *p* = 0.724; FFM, *p* = 0.213; LBM, *p* = 0.140). These findings indicate no evidence of small-study effects or publication bias (see Figure 6).

### 3.9 Certainty of evidence (GRADE)

The certainty of evidence for the primary outcomes is summarized in Table 2. High-certainty evidence was found for Wingate peak power, based on consistent and precise results across studies. Moderate-certainty evidence was observed for squat 1RM, FFM, LBM, and Wingate mean power, primarily due to moderate inconsistency or imprecision between studies. Evidence for leg press 1RM and CMJ was rated as low, due to serious imprecision in leg press 1RM and significant inconsistency compounded by imprecision in CMJ outcomes. These downgrades reflect variability in study designs, measurement protocols, and reporting practices, which affected the reliability of the effect estimates.

**Table 2:** Certainty of evidence (GRADE framework) for primary outcomes.

Outcome	Participants (RCTs)	Risk of bias	Inconsistency	Indirectness	Imprecision	Publication bias	Effect estimate (MD (95% CI))	Overall certainty
Squat 1RM	300 (16 RCTs)	Not serious	Serious	Not serious	Not serious	None detected	11.9 (7.60, 16.20)	⨁⨁⨁◯ Moderate
Leg press 1RM	273 (12 RCTs)	Not serious	Serious	Not serious	Very serious	None detected	3.58 (−7.85, 15.01)	⨁⨁◯ Low
FFM	327 (15 RCTs)	Not serious	Not serious	Not serious	Not serious	None detected	2.32 (0.76, 3.89)	⨁⨁⨁◯ Moderate
LBM	344 (16 RCTs)	Not serious	Serious	Not serious	Not serious	None detected	1.84 (0.53, 3.16)	⨁⨁⨁◯ Moderate
Wingate peak power	246 (12 RCTs)	Not serious	Not serious	Not serious	Not serious	None detected	71.27 (38.09, 104.45)	⨁⨁⨁⨁ High
Wingate mean power	218 (11 RCTs)	Not serious	Serious	Not serious	Not serious	None detected	39.69 (15.83, 63.56)	⨁⨁⨁ Moderate
CMJ	273 (11 RCTs)	Not serious	Very serious	Not serious	Serious	None detected	2.70 (0.18, 5.21)	⨁⨁◯◯ Low

MD, mean difference. Certainty of evidence was assessed using GRADE for continuous outcomes. For CMJ, certainty was downgraded due to very high heterogeneity (*I*^2^ = 89%) and variability in testing protocols.

## 4 Discussion

### 4.1 Main findings

This systematic review and meta-analysis included 37 randomized controlled trials in healthy men aged 18–30 years and examined whether training context modifies the effects of creatine supplementation. Training context (RT vs. non-RT) was prespecified as the primary analytical framework to assess contextual dependence. Lean mass outcomes (FFM and LBM) increased significantly only when creatine was combined with RT, whereas no significant effects were observed in non-RT settings. In contrast, Wingate peak and mean power improved significantly in both contexts, with little evidence that training type moderated these outcomes. CMJ showed a small overall improvement, but the high heterogeneity (*I*^2^ = 89%) warrants a cautious interpretation, as it limits the robustness and stability of this pooled estimate. Training context did not significantly moderate this effect. For maximal strength, squat 1RM improved in both contexts, whereas leg press 1RM showed no significant overall effect.

### 4.2 Comparison with previous evidence

The present findings are broadly consistent with prior meta-analyses reporting positive effects of creatine supplementation on strength and anaerobic performance (67). Additionally, the results extend this literature by explicitly testing training context as a prespecified moderator within a narrowly defined population. Previous reviews have frequently pooled mixed-sex samples and wide age ranges and have often examined training modality only through *post hoc* subgroup analyses (11). By restricting inclusion to healthy young men and stratifying analyses *a priori* by RT and non-RT, the current study enhances interpretability and translational relevance.

For body composition, the observed increases in FFM and LBM under RT conditions align with earlier syntheses reporting modest lean mass gains with creatine supplementation (15). However, the absence of significant effects in non-RT settings highlights the importance of a sufficiently robust resistance-based stimulus for translating creatine’s metabolic effects into measurable changes in lean mass (2). It should also be noted that increases in FFM or LBM, particularly in shorter-duration interventions, may partly reflect creatine-induced intracellular water retention rather than solely contractile tissue accretion.

With respect to anaerobic performance, the consistent improvements in Wingate peak and mean power across both training contexts support the view that creatine’s ergogenic effects on short-duration, high-intensity output are relatively independent of training modality (68). These findings are consistent with the established role of the phosphocreatine system in rapid ATP resynthesis and indicate that concurrent RT is not required to realize improvements in anaerobic power (2).

### 4.3 Interpretation and potential mechanisms

RT is mechanistically distinct because it provides planned progressive overload with high mechanical tension and sufficient training volume to reach hypertrophy-relevant thresholds. In contrast, non-RT modalities are more heterogeneous, and the magnitude and distribution of these stimuli across target muscles are often less consistent. The outcome-specific pattern of effects likely reflects complementary acute and chronic pathways. Increased intramuscular phosphocreatine availability likely contributes to improvements in short maximal efforts such as the Wingate test. By comparison, body composition changes are more indicative of longer-term adaptations that may be facilitated by greater training load and recovery capacity during RT.

Under non-RT conditions, training stimuli are more heterogeneous in terms of intensity, loading patterns, and targeted musculature, which may limit the extent of any creatine-related metabolic advantages being translated into consistent increases in lean mass (69). Accordingly, creatine appears to function as a training stimulus amplifier rather than an independent driver of increases in FFM or LBM (70).

The high variance noted in CMJ outcomes likely reflects that CMJ is a multi-factorial, skill-dependent performance outcome. CMJ height depends not only on energy availability but also on maximal strength (71), neuromuscular coordination, stretch–shortening cycle efficiency, technical execution, and testing protocols (72–74). Accordingly, pooled CMJ effects should be interpreted with caution, even when statistically significant.

The larger effect size for squat one-repetition maximum observed in the non-RT subgroup should be interpreted cautiously. This subgroup included a limited number of studies and participants, and differences in baseline training status, technical learning effects, and testing familiarity may have contributed (77, 78). This finding should not be interpreted as evidence that non-RT contexts are superior for strength development with creatine supplementation. In addition, strength improvements observed under non-RT conditions may reflect testing specificity, neural adaptations, or learning effects rather than true hypertrophic adaptations. In the absence of a structured resistance-based hypertrophic stimulus, increases in 1RM may predominantly reflect neuromuscular or task-specific adaptations rather than measurable changes in muscle mass.

### 4.4 Practical implications

For healthy young men seeking to increase lean mass, the present findings support the combined use of creatine supplementation with structured RT (2). In the absence of an adequate resistance-based stimulus, creatine alone should not be expected to produce reliable improvements in body composition (11).

Improvements in short-duration anaerobic power may be achieved across a range of training modalities (79), supporting the use of creatine in both RT and non-RT contexts when anaerobic performance is a primary goal (8).

For practitioners looking to enhance CMJ performance, the usage of creatine should be viewed as an adjunct in well-designed explosive or strength-oriented training programs that are undertaken over a sufficient duration of time (22). Therefore, emphasis should be placed on training quality and standardization of protocols over short-term supplementation only (71, 80). A summary of these outcome-specific patterns is provided in Table 3.

**Table 3:** Summary of outcome-specific effects by training context (RT vs. non-RT).

Outcome	RT context	Non-RT context	Between-subgroup difference	Practical takeaway
FFM	Significant increase	Not significant	Significant (*p* = 0.009)	Lean mass goals: creatine + RT
LBM	Significant increase	Not significant	Significant (*p* < 0.001)	Lean mass goals: creatine + RT
Squat 1RM	Significant increase	Significant increase^a^	Significant (*p* = 0.022)	Interpret non-RT gains cautiously
Wingate peak power	Significant increase	Significant increase	Not significant (*p* = 0.236)	Anaerobic goals: effective across modalities
Wingate mean power	Significant increase	Significant increase	Not significant (*p* = 0.902)	Anaerobic goals: effective across modalities
CMJ	Not significant	Pooled improvement^b^ (non-RT subgroup)	Not significant (*p* = 0.068)	Skill-dependent; interpret cautiously

FFM, fat-free mass; LBM, lean body mass; RT, resistance training; CMJ, countermovement jump; 1RM, one-repetition maximum. Significant/not significant refers to the pooled meta-analytic effect (*p* < 0.05) within each training-context subgroup.

a Larger effect size observed in the non-RT subgroup, but this is based on limited studies and participants (see Section 4.3).

b High heterogeneity (*I*^2^ = 88.5%) warrants cautious interpretation of the CMJ finding.

### 4.5 Strengths, limitations, and future directions

Key strengths of this review include its focus on a narrowly defined population, the prespecified use of training context as the primary analytical framework, and the synthesis of strength, performance, and body composition outcomes within a single analysis. Limitations include the heterogeneity of non-RT training modalities, the limited number of studies in some subgroups, and the predominance of short- to medium-term interventions. Future research should include longer-duration trials, more granular classification of training stimuli, and standardized assessment protocols, particularly for explosive performance outcomes.

## 5 Conclusion

In healthy men aged 18–30 years, creatine supplementation produced outcome-specific effects that were partially dependent on training context. Lean mass outcomes (FFM and LBM) increased only when creatine was combined with RT, whereas anaerobic power (Wingate peak and mean power) improved in both RT and non-RT settings. Squat 1RM improved in both settings, leg press 1RM showed no significant overall effect, and CMJ showed a small pooled improvement with substantial heterogeneity and uncertain moderation by training context. This *a priori* training-context framework provides more direct evidence regarding the contextual dependence of creatine’s effects and is consistent with pairing creatine with RT when lean mass gain is the primary goal.

## Data Availability

The original contributions presented in the study are included in the article/Supplementary material, further inquiries can be directed to the corresponding author.
