# Correction: Creatine supplementation in young men under resistance versus non-resistance training: a systematic review and meta-analysis of strength, performance, and lean mass

**DOI:** 10.3389/fnut.2026.1854605

**Published:** 2026-05-20

**Authors:** Jinfa Gu, Yan Li, Jialing Xiao, Yu Zhang

**Affiliations:** 1 School of Stomatology, Qilu Medical University, Zibo, China; 2 Exercise & Sports Science Programme, School of Health Sciences, Universiti Sains Malaysia, Kubang Kerian, Kelantan, Malaysia; 3 Department of Pharmacy, Ezhou Central Hospital, Ezhou, Hubei, China; 4 College of Nursing and Health, Jiujiang Polytechnic University of Science and Technology, Gongqingcheng, China

**Keywords:** anaerobic performance, body composition, creatine, meta-analysis, resistance training, supplementation

In the published article, two studies (Ostojic et al., 2004 and Percário et al., 2012) were inadvertently included despite having participants outside the prespecified eligible age range of 18–30 years. These studies have been removed, and the affected PRISMA flow diagram, study-characteristics table, risk-of-bias summary, outcome analyses, subgroup analyses, GRADE table, figures, and supplementary files have been updated accordingly.

Following these corrections, the main conclusions remain largely stable: creatine supplementation appears to enhance lean mass primarily when combined with resistance training, and anaerobic performance outcomes, including Wingate outcomes, improve across training contexts. Some secondary findings have been refined; in particular, the CMJ pooled estimate has changed from 2.87 to 2.70 cm, remains highly heterogeneous, and no longer supports a clear interpretation regarding consistency in longer-duration interventions. Details of the corrected sections are listed below.

In the published article, references 52 (Ostojic et al., 2004) and 54 (Percário et al., 2012) were inadvertently included. The reference list has been updated to remove these references. The corresponding intext citations have also been removed from the article body.

In the abstract, the number of included trials, and the pooled CMJ effect estimate and heterogeneity value, were reported incorrectly. The **Methods** and **Results** sections of the abstract have been corrected to read:

**“Methods:** This systematic review and meta-analysis pooled RCT evidence in healthy men aged 18–30 years old to quantify the effects of creatine supplementation in terms of body composition, maximal strength, and exercise performance. All the databases were searched up to 1 October 2025, and 37 eligible trials were discovered. The context of training was prespecified—RT vs. non-RT, and used as the main comparison. Pooled estimates were made using random effects models for FFM, LBM, and Wingate peak and mean power, CMJ, and 1RM outcomes. The exploratory subgroup analyses were done to investigate whether training condition and intervention duration moderated the effects.”

**“Results:** The number of trials that were considered according to the inclusion criteria was 37. When using RT, creatine supplementation led to significant gains in FFM (+3.39 kg) and LBM (+2.70 kg), but not to significant gains in non-RT conditions. Wingate peak and mean power both increased in both contexts (peak power +71.27 W; mean power +39.69 W), with no evidence that training context modified these results. CMJ showed a pooled improvement of 2.70 cm; however, this estimate should be interpreted with caution due to high heterogeneity (*I*^2^ = 89%). Exploratory subgroup analyses by intervention duration should be interpreted cautiously because of the small number of studies and high heterogeneity.”

A correction has been made to **Results**, *3.1 Study Selection, Paragraph 1*. The paragraph previously reported incorrect counts for excluded full-text reports and included studies and did not report the exclusion of studies with participants outside the eligible age range. The corrected paragraph appears below:

“A total of 2,472 records were identified through database searching. After removal of 1,449 duplicates, 1,023 records were screened, of which 909 were excluded. Full-text reports were sought for 114 records; six reports could not be retrieved, leaving 108 reports assessed for eligibility. Of these, 71 reports were excluded for the following reasons: lack of an eligible control group (*n* = 26), absence of prespecified outcomes (*n* = 16), insufficient data to calculate effect sizes (*n* = 19), inappropriate study design (*n* = 8), and participants outside the eligible age range (*n* = 2). Ultimately, 37 studies were included in the review (see Figure 1).”

A correction has been made to **Results**, *3.2 Study Characteristics, Paragraph 1*. The paragraph previously reported incorrect counts of included studies, subgroup distribution, study list, age range, and percentage of RT studies. The corrected paragraph appears below:

“A total of 37 such RCTs were included, consisting of 24 studies in RT settings and 13 in non-RT settings (Ahmun et al., 2005; Arciero et al., 2001; Becque et al., 2000; Bemben et al., 2001; Bonilla et al., 2021; Camic et al., 2014; Cribb et al., 2007; del Favero et al., 2012; Earnest et al., 1995; Griffen et al., 2015; Havenetidis and Bourdas, 2003; Herda et al., 2009; Hoffman et al., 2006; Izquierdo et al., 2002; Javierre et al., 2004; Kaviani et al., 2019; Kelly and Jenkins, 1998; Kilduff et al., 2002; Law et al., 2009; Mujika et al., 2000; Noonan et al., 1998; Nunes et al., 2017; Peeters et al., 1999; Saremi et al., 2010; Stone et al., 1999; Stout et al., 1999; Syrotuik et al., 2000; Taylor et al., 2011; Trexler et al., 2016; van Loon et al., 2003; Volek et al., 1999; Volek et al., 2004; Wang et al., 2018; Wang et al., 2016; Wilder et al., 2002; Willoughby and Rosene, 2001; Zuniga et al., 2012). Not all outcomes were reported in some studies, but in every study, at least one prespecified outcome was noted. Mean ages were between 18.5 and 29.5 years, and training status ranged from sedentary or untrained to well-trained, competitive, and elite athletes. RT was the most common modality (about 65% of studies), with other protocols that included team sports (rugby or American football, soccer, handball, and basketball), individual sports (canoeing or rowing and sprinting), cycling-based protocols (including Wingate testing), and those related to strength- or power-related performance assessment.”

A correction has been made to **Results**, *3.3 Risk of Bias Results, Paragraph 1*. The paragraph previously reported incorrect trial counts, percentages, and outcome-measurement counts. The corrected paragraph appears below:

“Risk of bias was evaluated using the Cochrane RoB 2 tool. Among the 37 trials, 25 (67.6%) were deemed to be at low risk of bias, nine (24.3%) raised some concerns, and three (8.1%) were considered to be at high risk. Some concerns most commonly arose from deviations from intended interventions (D2), with fewer studies flagged for missing outcome data (D3) or selective reporting (D5). Outcome measurement (D4) was regarded as low risk in nearly all trials (36/37), consistent with predominantly objective performance and body-composition outcomes (see Figure 2).”

A correction has been made to **Results**, *3.4 Overall effects, Paragraph 2*. The sentence previously reported an incorrect CMJ overall pooled estimate, study count, participant count, *p* value, and heterogeneity. The corrected sentence appears below:

“For countermovement jump, the pooled effect favored creatine supplementation (11 studies, *N* = 273; MD = 2.70 cm, 95% CI: 0.18–5.21; *p* = 0.04; *I*^2^ = 89%).”

A further correction has been made to **Results**, *3.4 Overall effects, Paragraph 3*. The sentence previously reported incorrect LBM overall pooled estimate, study count, participant count, *p* value, and heterogeneity. The corrected sentence appears below:

“Significant pooled effects favoring creatine supplementation were also observed for FFM (15 studies, *N* = 327; MD = 2.32 kg, 95% CI 0.76 to 3.89; *p* = 0.004; *I*^2^ = 13.6%) and LBM (16 studies, *N* = 344; MD = 1.84 kg, 95% CI 0.53 to 3.16; *p* = 0.006; *I*^2^ = 37%).”

A correction has been made to **Results**, *3.5 Subgroup analysis by training context (RT versus non-RT), Paragraph 2*. The sentence previously reported incorrect non-RT study count, participant count, pooled estimate, and between-subgroup statistics. The corrected sentence appears below:

“For countermovement jump, the pooled effect was not significant in RT studies (five studies, *N* = 88; MD = 4.19, 95% CI −1.28 to 9.66; *p* = 0.13; *I*^2^ = 95%), whereas no significant pooled effect was observed in non-RT studies (six studies, *N* = 185; MD = 1.57, 95% CI −0.26 to 3.40; *p* = 0.09; *I*^2^ = 43%); the between-subgroup difference did not reach conventional statistical significance (*p* = 0.37; *I*^2^ = 0%; see Figure 4).”

A further correction has been made to **Results**, *3.5 Subgroup analysis by training context (RT versus non-RT), Paragraph 4*. The sentence previously reported incorrect LBM subgroup study counts, participant counts, pooled estimates, and between-subgroup statistics. The corrected sentence appears below:

“For body composition outcomes, a significant pooled effect was observed for FFM in RT studies (12 studies, *N* = 228; MD = 3.39 kg, 95% CI 1.77 to 5.02; *p* < 0.001; *I*^2^ = 0%), but not in non-RT studies (three studies, *N* = 99; MD = −0.89 kg, 95% CI −3.94 to 2.15; *p* = 0.566; *I*^2^ = 24.0%), with evidence of a between-subgroup difference (*p* = 0.009; *I*^2^ = 85.4%). LBM showed a significant pooled effect in RT studies (12 studies, *N* = 279; MD = 2.70 kg, 95% CI 1.56 to 3.85; *p* = 0.0001; *I*^2^ = 0%), whereas no significant pooled effect was observed in non-RT studies (four studies, *N* = 65; MD = −0.54 kg, 95% CI −1.75 to 0.68; *p* = 0.39; *I*^2^ = 0%); the between-subgroup difference was significant (*p* = 0.0001; *I*^2^ = 93.1%).”

A correction has been made to **Results**, *3.6 Subgroup analyses for CMJ, Paragraph 1*. The sentence previously reported incorrect study counts, participant counts, pooled estimates, and subgroup statistics by intervention duration. The corrected sentence appears below:

“When stratified by intervention duration, the pooled effect was not significant in studies lasting < 8 weeks (eight studies, *N* = 225; MD = 0.77 cm, 95% CI −0.97 to 2.50; *p* = 0.39; *I*^2^ = 67%), whereas a significant pooled effect was observed in studies lasting ≥8 weeks (three studies, *N* = 48; MD = 8.06 cm, 95% CI 3.87 to 12.25; *p* = 0.0002; *I*^2^ = 79%); a between-subgroup difference was observed (*p* = 0.002; *I*^2^ = 89.9%; see Figure 5).”

A further correction has been made to **Results**, *3.6 Subgroup analyses for CMJ, Paragraph 2*. The sentence previously reported incorrect study counts, participant counts, pooled estimates, and subgroup statistics by competitive level. The corrected sentence appears below:

“When stratified by competitive level, a significant pooled effect was observed in competitive participants (seven studies, *N* = 138; MD = 3.36 cm, 95% CI 0.10 to 6.63; *p* = 0.04; *I*^2^ = 93%), whereas no significant pooled effect was observed in recreational participants (four studies, *N* = 135; MD = 1.17 cm, 95% CI −1.77 to 4.12; *p* = 0.44; *I*^2^ = 39%); no significant between-subgroup difference was detected (*p* = 0.33; *I*^2^ = 0%).”

A further correction has been made to **Results**, *3.6 Subgroup analyses for CMJ, Paragraph 3*. The sentence previously reported incorrect study counts, participant counts, pooled estimates, and subgroup statistics by supplementation frequency. The corrected sentence appears below:

“When stratified by supplementation frequency, no significant pooled effect was observed in studies with daily supplementation (eight studies, *N* = 157; MD = 2.81 cm, 95% CI −0.22 to 5.83; *p* = 0.07; *I*^2^ = 92%), whereas no significant pooled effect was observed in studies with non-daily supplementation (three studies, *N* = 116; MD = 2.52 cm, 95% CI −0.53 to 5.57; *p* = 0.11; *I*^2^ = 9%); no evidence of a between-subgroup difference was observed (*p* = 0.90; *I*^2^ = 0%).”

A correction has been made to **Results**, *3.8 Assessment of publication bias, Paragraph 1*. The sentence previously reported incorrect Egger's test *p* values for CMJ and LBM. The corrected sentence appears below:

“Visual inspection of funnel plots revealed no clear asymmetry, and Egger's tests were non-significant across outcomes (squat 1RM, *p* = 0.915; leg press 1RM, *p* = 0.397; CMJ, *p* = 0.949; Wingate peak power, *p* = 0.340; Wingate mean power, *p* = 0.724; FFM, *p* = 0.213; LBM, *p* = 0.140). These findings indicate no evidence of small-study effects or publication bias (see Figure 6).”

A correction has been made to **Discussion**, *4.1 Main findings, Paragraph 1*. The paragraph previously reported an incorrect total number of included trials and an outdated CMJ heterogeneity value. The corrected paragraph appears below:

“This systematic review and meta-analysis included 37 randomized controlled trials in healthy men aged 18–30 years and examined whether training context modifies the effects of creatine supplementation. Training context (RT vs. non-RT) was prespecified as the primary analytical framework to assess contextual dependence. Lean mass outcomes (FFM and LBM) increased significantly only when creatine was combined with RT, whereas no significant effects were observed in non-RT settings. In contrast, Wingate peak and mean power improved significantly in both contexts, with little evidence that training type moderated these outcomes. CMJ showed a small overall improvement, but the high heterogeneity (*I*^2^ = 89%) warrants a cautious interpretation, as it limits the robustness and stability of this pooled estimate. Training context did not significantly moderate this effect. For maximal strength, squat 1RM improved in both contexts, whereas leg press 1RM showed no significant overall effect.”

A correction has been made to **Discussion**, *4.3 Interpretation and potential mechanisms, Paragraph 3*. A sentence referring to more consistent CMJ effects in longer interventions has been removed to align the discussion with the revised subgroup results. The corrected paragraph appears below:

“The high variance noted in CMJ outcomes likely reflects that CMJ is a multi-factorial, skill-dependent performance outcome. CMJ height depends not only on energy availability but also on maximal strength (73), neuromuscular coordination, stretch–shortening cycle efficiency, technical execution, and testing protocols (74–76). Accordingly, pooled CMJ effects should be interpreted with caution, even when statistically significant.”

There was a mistake in Figure 1 as published. The PRISMA flow diagram contained incorrect counts for excluded reports and included studies. The corrected Figure 1 appears below.

**Figure 1:**
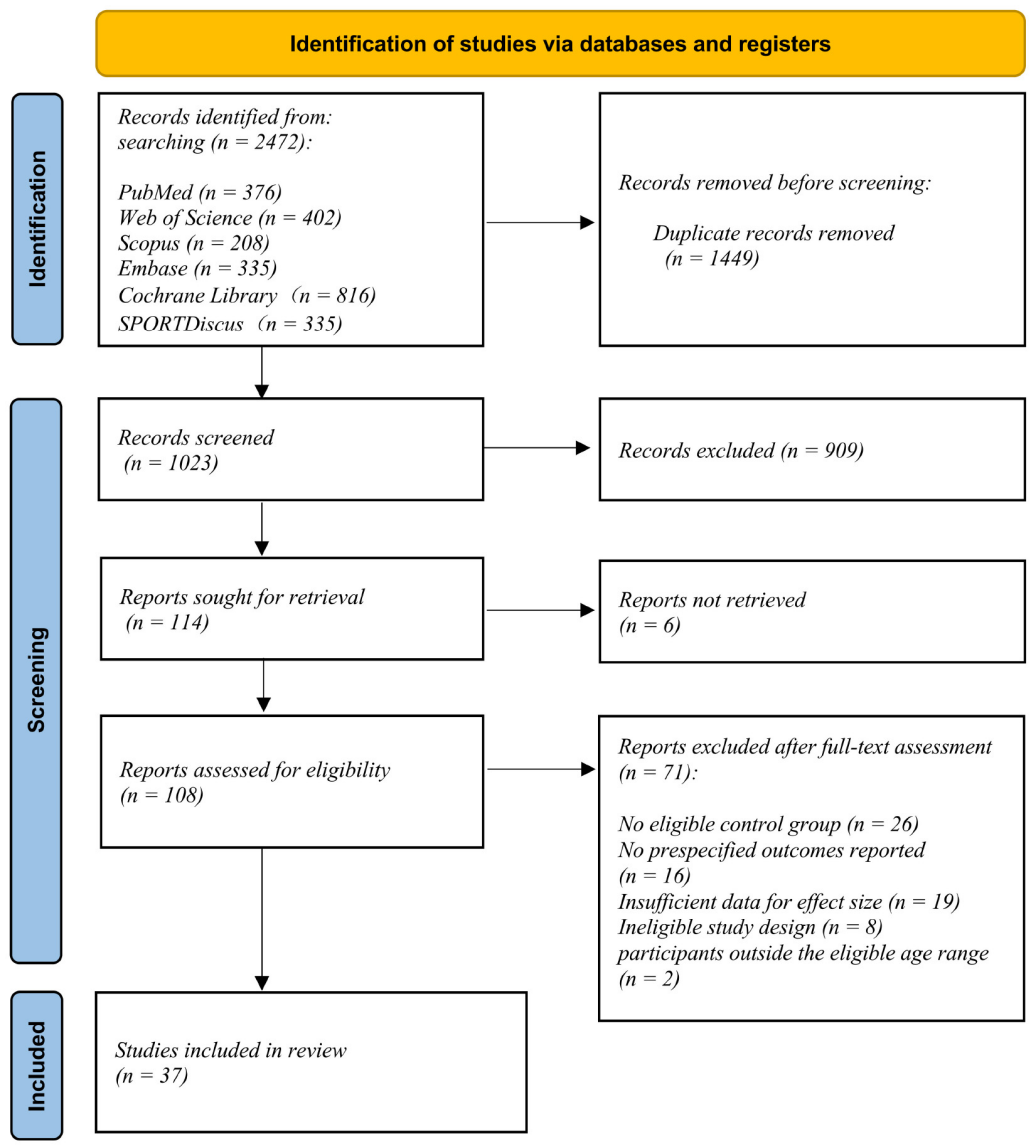
PRISMA flow diagram illustrating the study selection process for the systematic review and meta-analysis.

There was a mistake in Figure 2 as published. The risk-of-bias figure was based on the original study set and has been updated accordingly. The corrected Figure 2 appears below.

**Figure 2:**
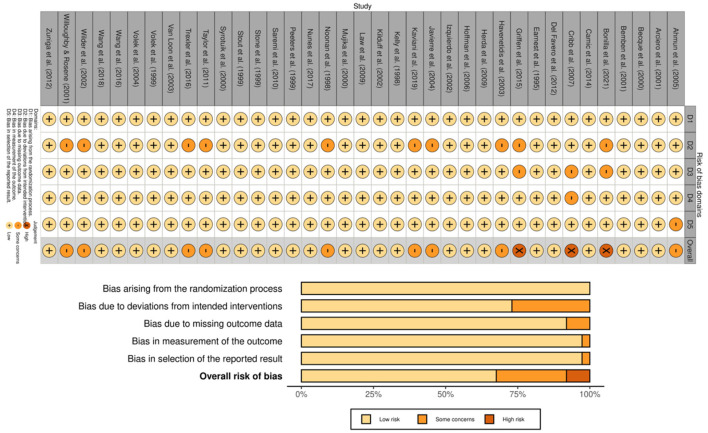
Risk-of-bias assessment of included trials using the Cochrane RoB 2 tool.

There was a mistake in Figure 3 as published. The pooled results shown for CMJ and LBM were based on the original study set and have been updated accordingly. The corrected Figure 3 appears below.

**Figure 3:**
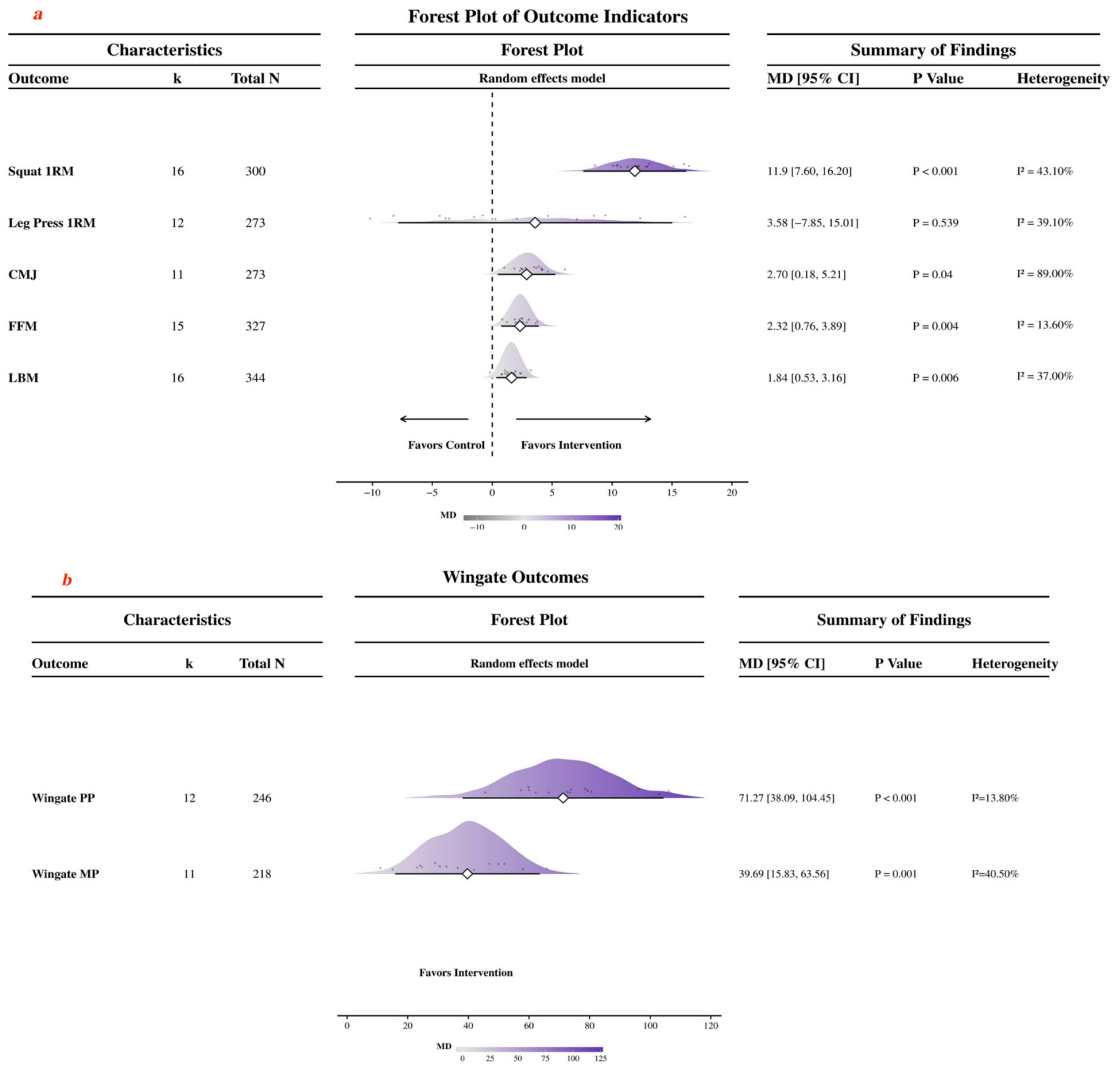
Forest plot of the effects of creatine supplementation on maximal strength, power, anaerobic performance, and body composition. **(a)** Forest plots for squat 1RM, leg press 1RM, CMJ, FFM, and LBM outcomes. **(b)** Forest plots for Wingate peak power and mean power outcomes.

There was a mistake in Figure 4 as published. The subgroup forest plot contained outdated subgroup results and has been updated accordingly. The corrected Figure 4 appears below.

**Figure 4:**
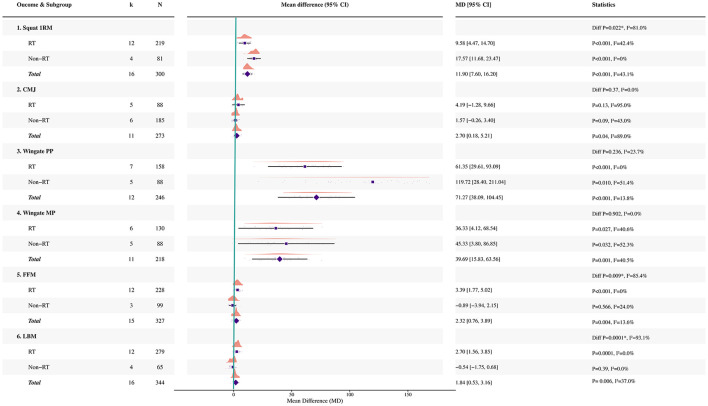
Forest plot of subgroup analyses stratified by RT and non-resistance training (non-RT).

There was a mistake in Figure 5 as published. The CMJ subgroup figure contained outdated subgroup results and has been updated accordingly. The corrected Figure 5 appears below.

**Figure 5:**
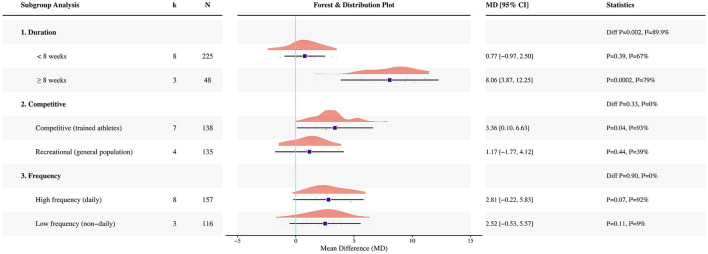
Forest plot of subgroup analyses for CMJ.

There was a mistake in Figure 6 as published. The funnel plots contained outdated publication bias results and have been updated accordingly. The corrected Figure 6 appears below.

**Figure 6:**
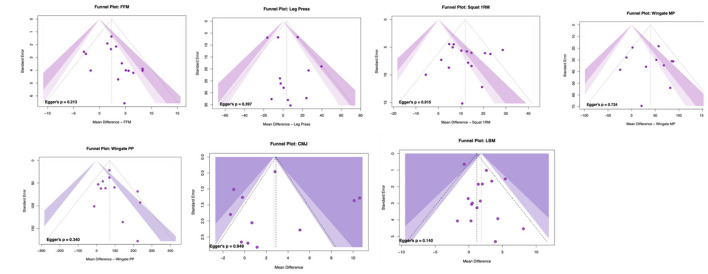
Funnel plots assessing publication bias.

There was a mistake in Figure A1 as published. The outcome-specific forest plots for CMJ and LBM were based on the original study set and have been updated accordingly. The corrected Figure A1 appears below.

**Figure A1:**
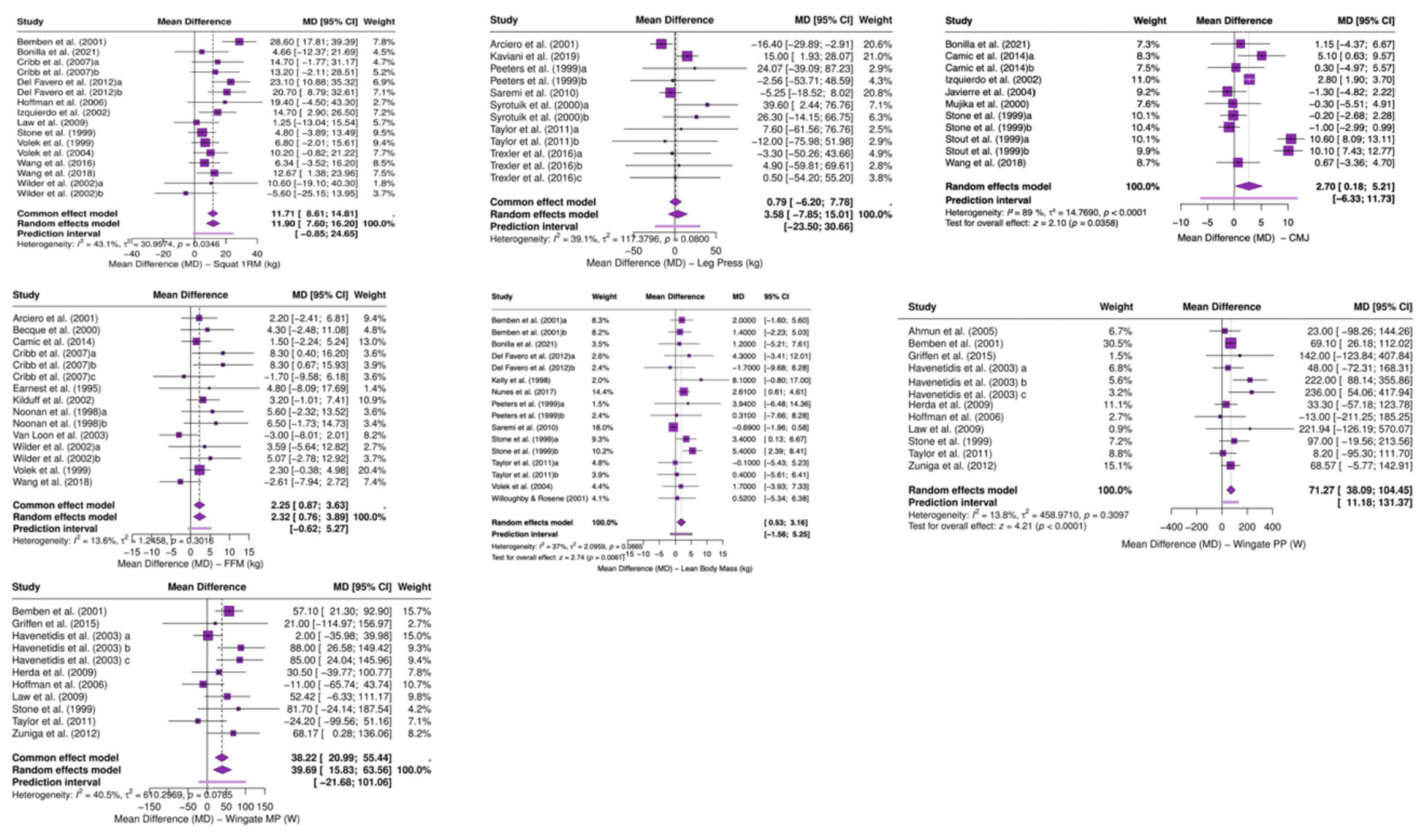
Outcome-specific forest plots for squat 1RM, leg press 1RM, CMJ, Wingate peak power, Wingate mean power, FFM, and LBM.

There was a mistake in Table 1 as published. The table included studies with participants outside the eligible age range and has been updated accordingly. The corrected Table 1 appears below.

**Table 1:** Characteristics of included studies.

Study citation	Mean age (years)	Sport/exercise modality	Subject level	Intervention protocol (dosage and frequency)	Duration	Control	Outcome measures
Ahmun et al. (31)	20.6	Rugby union	Highly trained	20 g/d (4 × 5 g)	5 days	Dextrose	Wingate power
Arciero et al. (32)	21	Resistance training	Healthy active (resistance-untrained)	20 g/d (5 days) + 10 g/d (23 days)	28 days	Dextrose	1RM leg press, FFM
Becque et al. (33)	21.5	Arm flexor strength training	≥1 year of weight training experience	Loading: 20 g/d (5 g × 4) for 5 days; maintenance: 2 g/d	6 weeks	Flavored sucrose drink	FFM
Bemben et al. (34)	19.2	American football	NCAA Division I athletes (redshirt)	20 g/d (5 days) + 5 g/d (maintenance)	9 weeks	Glucose	1RM squat, Wingate power, LBM
Bonilla et al. (35)	26.6	Cluster-set Resistance Training	Resistance-trained (>2 years experience)	0.1 g/kg/d (post-workout)	8 weeks	Protein	1RM squat, CMJ, LBM
Camic et al. (36)	22.1	Anaerobic performance tests	Untrained (in resistance exercise)	1.25 or 2.50 g/d PEG-creatine (1 dose/d)	28 days	Cellulose	CMJ, FFM
Cribb et al. (37)	24	Bodybuilding	Recreational male bodybuilders	1.5 g/kg/d supplement (~0.3 g/kg load + 0.1 g/kg Maint)	11 weeks	Glucose	1RM squat, FFM
Del Favero et al. (38)	24	Strength/power tests	Untrained (not engaged in RT)	20 g/d (2 x 10 g)	10 days	Dextrose	1RM squat, LBM
Earnest et al. (39)	29.5	Bench press and Wingate bike tests	~11 years of training experience	20 g/d (5 g × 4) during the supplementation period	28 days	Glucose placebo	FFM
Griffen et al. (40)	21.6	Cycling (Wingate)	Well-trained men	20 g/d (4 × 5 g)	7 days	Placebo	Wingate power
Havenetidis et al. (41)	29.4	Cycling (wingate)	Sprint trained males	10–35 g/d (divided doses)	4 days	Placebo	Wingate power
Herda et al. (42)	21	Resistance exercise/Wingate	Recreationally active	5 g/d (1 dose/d)	30 days	Placebo	Wingate power
Hoffman et al. (43)	19	American football	Collegiate athletes	10.5 g/d (2 doses/d)	10 weeks	Placebo	1RM squat, Wingate power
Izquierdo et al. (44)	22	Handball	Trained athletes	20 g/d (4 × 5 g)	5 days	Placebo	1RM squat, CMJ
Javierre et al. (45)	22.9	Running (sprints)	Physically active	20 g/d (4 × 5 g)	5 days	Placebo	CMJ
Kaviani et al. (46)	23	Resistance training	Sedentary/inactive	0.07 g/kg/d (2 doses/d)	8 weeks	Placebo	1RM leg press
Kelly et al. (47)	26.8	Powerlifting	Competitive	20 g/d (load) + 5 g/d (Maint)	26 days	Glucose	LBM
Kilduff et al. (48)	24	Isometric bench-press	At least 2 years of structured training experience	20 g/d (10 g × 2) mixed with 180 g dextrose for 5 days	5 days	200 g/d glucose polymer	FFM
Law et al. (49)	23.1	Basketball	Trained athletes	20 g/d (4 × 5 g)	5 days	Maltodextrin	1RM squat, Wingate power
Mujika et al. (50)	20.3	Soccer	Highly trained	20 g/d (4 × 5 g)	6 days	Maltodextrin	CMJ
Noonan et al. (51)	19.4	Weight training and speed drills	NCAA Division II football team	Loading: 20 g/d (5 g × 4) for 5 days; maintenance: 100 or 300 mg/kg FFM	8 weeks	Dextrose placebo	FFM
Nunes et al. (14)	22.7	Resistance training	Resistance trained	0.3 g/kg/d (load) + 0.03 g/kg/d (Maint)	8 weeks	Maltodextrin	LBM
Peeters et al. (53)	21.2	Resistance training	Experienced (>2 years)	20 g/d (load) + 10 g/d (Maint)	6 weeks	Maltodextrin	1RM leg press, LBM
Saremi et al. (55)	23.4	Resistance training	Healthy untrained	0.3 g/kg/d (load) + 0.05 g/kg/d (Maint)	8 weeks	Cellulose	1RM leg press, LBM
Stone et al. (56)	18.5	American football	Collegiate athletes	0.22 g/kg/d (3 doses/d)	5 weeks	Placebo (silica)	1RM squat, CMJ, Wingate power, LBM
Stout et al. (57)	19.6	American football	Collegiate athletes	21 g/d (load) + 10.5 g/d (Maint)	8 weeks	Glucose (CHO)	CMJ
Syrotuik et al. (58)	22.1	Resistance training	Resistance trained	0.3 g/kg/d (load) + 0.03 g/kg/d (Maint)	37 days	Placebo	1RM leg press
Taylor et al. (59)	21.3	Resistance training	Resistance trained	5 g/d (1 dose/d)	8 weeks	Placebo	1RM leg press, Wingate power, LBM
Trexler et al. (60)	21.2	Resistance training	Recreationally trained	5 g/d (1 dose/d)	28 days	Placebo	1RM leg press
Van Loon et al. (61)	20.6	Repeated supramaximal sprint and endurance cycling	No history of regular exercise training	Loading: 20 g/d for 5 days; maintenance: 2 g/d for 37 days	6 weeks	Placebo without creatine	FFM
Volek et al. (62)	23.4	Resistance training	Resistance trained	25 g/d (load) + 5 g/d (Maint)	12 weeks	Placebo	1RM squat, FFM
Volek et al. (63)	20.3	Resistance training (overreaching)	Resistance trained	0.3 g/kg/d (Load) + 0.03 g/kg/d (Maint)	4-5 weeks	Placebo	1RM squat, LBM
Wang et al. (65)	21.1	Complex training (squat/jump)	University athletes	20 g/d (load) + 2 g/d (Maint)	30 days	CMC	1RM squat
Wang et al. (64)	20.2	Kayak/canoe	University athletes	20 g/d (4 × 5 g)	6 days	CMC	1RM squat, CMJ, FFM
Wilder et al. (66)	19.3	American football	Collegiate athletes	20 g/d (load) + 5 g/d (Maint)	10 weeks	Glucose polymers	1RM squat, FFM
Willoughby and Rosene (67)	20.4	Resistance training	Untrained males	6 g/d (1 dose/d)	12 weeks	Dextrose	LBM
*Zuniga et al. (68)*	*22.5*	*Resistance training*	*Resistance trained*	*20 g/d (4 × 5 g)*	*7 days*	*Maltodextrin*	*Wingate power*

1RM, one-repetition maximum; CMJ, countermovement jump; FFM, fat-free mass; LBM, lean body mass; RT, resistance training; CHO, carbohydrate; PEG, polyethylene glycol (PEG-creatine); CMC, carboxymethylcellulose; g/d, grams/day; g/kg/d, grams/kg/day. Loading and maintenance denote high-dose initiation followed by a lower-dose phase; post-workout indicates intake immediately after training.

There was a mistake in Table 2 as published. The GRADE table contained outdated participant counts and effect estimates, and the table note for CMJ heterogeneity was updated accordingly. The corrected Table 2 appears below.

**Table 2:** Certainty of evidence (GRADE framework) for primary outcomes.

Outcome	Participants (RCTs)	Risk of bias	Inconsistency	Indirectness	Imprecision	Publication bias	Effect estimate (MD (95% CI))	Overall certainty
Squat 1RM	300 (16 RCTs)	Not serious	Serious	Not serious	Not serious	None detected	11.9 (7.60, 16.20)	⊕⊕⊕○ moderate
Leg press 1RM	273 (12 RCTs)	Not serious	Serious	Not serious	Very serious	None detected	3.58 (−7.85, 15.01)	⊕⊕○ low
FFM	327 (15 RCTs)	Not serious	Not serious	Not serious	Not serious	None detected	2.32 (0.76, 3.89)	⊕⊕⊕○ moderate
LBM	344 (16 RCTs)	Not serious	Serious	Not serious	Not serious	None detected	1.84 (0.53, 3.16)	⊕⊕⊕○ moderate
Wingate peak power	246 (12 RCTs)	Not serious	not serious	Not serious	Not serious	None detected	71.27 (38.09, 104.45)	⊕⊕⊕⊕ high
Wingate mean power	218 (11 RCTs)	Not serious	Serious	Not serious	Not serious	None detected	39.69 (15.83, 63.56)	⊕⊕⊕ moderate
CMJ	273 (11 RCTs)	Not serious	Very serious	Not serious	Serious	None detected	2.70 (0.18, 5.21)	⊕⊕○○ low

MD, mean difference. Certainty of evidence was assessed using GRADE for continuous outcomes. For CMJ, certainty was downgraded due to very high heterogeneity (I2 = 89%) and variability in testing protocols.

There was a mistake in Table 3 as published. The summary table contained outdated subgroup results for LBM and CMJ, and the table notes were updated accordingly. The corrected Table 3 appears below.

**Table 3:** Summary of outcome-specific effects by training context (RT vs. non-RT).

Outcome	RT context	Non-RT context	Between-subgroup difference	Practical takeaway
FFM	Significant increase	Not significant	Significant (*p* = 0.009)	Lean mass goals: creatine + RT
LBM	Significant increase	Not significant	Significant (*p* = 0.0001)	Lean mass goals: creatine + RT
Squat 1RM	Significant increase	Significant increase^a^	Significant (*p* = 0.022)	Interpret non-RT gains cautiously
Wingate peak power	Significant increase	Significant increase	Not significant (*p* = 0.236)	Anaerobic goals: effective across modalities
Wingate mean power	Significant increase	Significant increase	Not significant (*p* = 0.902)	Anaerobic goals: effective across modalities
CMJ	Not significant	Not significant	Not significant (*p* = 0.37)	Skill-dependent; interpret cautiously

FFM, fat-free mass; LBM, lean body mass; RT, resistance training; CMJ, countermovement jump; 1RM, one-repetition maximum. Significant/not significant refers to the pooled meta-analytic effect (p < 0.05) within each training-context subgroup.

aLarger effect size observed in the non-RT subgroup, but this is based on limited studies and participants (see Section 4.3).

The original version of this article has been updated.

