# Supplementary material for: Creatine supplementation in young men under resistance versus non-resistance training: a systematic review and meta-analysis of strength, performance, and lean mass
Source: Front Nutr. 2026 Apr 8;13:1800546. doi: 10.3389/fnut.2026.1800546 (PMC13099317; doi:10.3389/fnut.2026.1800546)
Supplement: Supplementary file 1 [file supplementary_file_1.docx]

**Detailed search strategies**

| **Database** | **Step** | **Search Query / Terms** |
| --- | --- | --- |
| **PubMed** | #1 | "creatine supplementation"[Title/Abstract] OR creatine[Title/Abstract] |
|  | #2 | randomized[Title/Abstract] OR randomised[Title/Abstract] OR placebo[Title/Abstract] |
|  | #3 | male[Title/Abstract] OR males[Title/Abstract] OR men[Title/Abstract] |
|  | #4 | training[Title/Abstract] OR exercise[Title/Abstract] |
|  | #5 | strength[Title/Abstract] OR power[Title/Abstract] OR jump*[Title/Abstract] OR "Wingate"[Title/Abstract] |
|  | **Final** | **#1 AND #2 AND #3 AND #4 AND #5** |
| **Web of Science** | #1 | TS=("creatine supplementation" OR creatine) |
|  | #2 | TS=(randomized OR randomised OR placebo) |
|  | #3 | TS=(male OR males OR men) |
|  | #4 | TS=(training OR exercise) |
|  | #5 | TS=(strength OR power OR jump* OR "Wingate") |
|  | **Final** | **#1 AND #2 AND #3 AND #4 AND #5** |
| **SPORTDiscus** | S1 | TI ("creatine supplementation" OR creatine) OR AB ("creatine supplementation" OR creatine) |
|  | S2 | TI (randomized OR randomised OR placebo) OR AB (randomized OR randomised OR placebo) |
|  | S3 | TI (male OR males OR men) OR AB (male OR males OR men) |
|  | S4 | TI (training OR exercise) OR AB (training OR exercise) |
|  | S5 | TI (strength OR power OR jump* OR "Wingate") OR AB (strength OR power OR jump* OR "Wingate") |
|  | **Final** | **S1 AND S2 AND S3 AND S4 AND S5** |
| **Embase** | #1 | (creatine OR "creatine supplementation").ti,ab. |
|  | #2 | (randomized OR randomised OR placebo).ti,ab. |
|  | #3 | (male OR males OR men).ti,ab. |
|  | #4 | (training OR exercise).ti,ab. |
|  | #5 | (strength OR power OR jump* OR Wingate).ti,ab. |
|  | **Final** | **#1 AND #2 AND #3 AND #4 AND #5** |
| **Cochrane Library** | #1 | "creatine supplementation" OR creatine:ti,ab,kw |
|  | #2 | randomized OR randomised OR placebo:ti,ab,kw |
|  | #3 | male OR males OR men:ti,ab,kw |
|  | #4 | training OR exercise:ti,ab,kw |
|  | #5 | strength OR power OR jump* OR "Wingate":ti,ab,kw |
|  | **Final** | **#1 AND #2 AND #3 AND #4 AND #5** |
